# Validation of the “purfling sign” for differentiating primary central nervous system lymphoma from high-grade glioma: a retrospective multicenter cohort study

**DOI:** 10.1186/s12880-026-02650-3

**Published:** 2026-08-10

**Authors:** Xiaofen Wu, Kuan Lu, Qi Guo, Chenchen Liu, Peng Ji, Dai Shi

**Affiliations:** 1 Department of Radiology, The Second Affiliated Hospital of Soochow University, Sanxiang Road No. 1055, Suzhou, Jiangsu 215004 China; 2 Department of Radiology, Second Hospital of Tianjin Medical University, Tianjin, 300041 China; 3 Department of MRI, Zhumadian Central Hospital, Zhumadian, Henan 463000 China

**Keywords:** Primary central nervous system lymphoma, Glioma, Purfling sign, Magnetic resonance imaging, Diagnostic performance

## Abstract

**Background:**

The study aimed to evaluate whether the imaging marker “purfling sign” can reliably distinguish primary central nervous system lymphoma (PCNSL) from high-grade glioma and systematically assess its diagnostic performance, differential utility and clinical value.

**Materials and methods:**

This retrospective multicenter cohort study included 209 patients with PCNSL and 285 patients with glioma who underwent contrast-enhanced magnetic resonance imaging (CE-MRI). Two board-certified neuroradiologists independently reviewed CE-MRI features including lesion number, “purfling sign”, tumor necrosis and cystic changes, and tumor parenchymal enhancement heterogeneity, with inter-rater reliability assessed using Cohen’s kappa coefficient. Group comparisons were performed between the PCNSL group and the glioma group, and the diagnostic performance of the “purfling sign” was evaluated by calculating its sensitivity, specificity, positive predictive value (PPV), negative predictive value (NPV), and diagnostic accuracy, with 95% confidence intervals (CIs) reported for all metrics.

**Results:**

PCNSL and glioma showed significant between-group differences in lesion number, tumor necrosis and cystic changes, and tumor parenchymal enhancement heterogeneity (all *P* < 0.001). The purfling sign also differed significantly between groups (*P* < 0.001): it was detected in 100 PCNSL cases (47.80%) and 34 glioma cases (11.93%), yielding a sensitivity of 47.85% (95% CI: 41.1%–54.6%), specificity of 88.07% (95% CI: 84.3%–91.8%), PPV of 74.63% (95% CI: 67.3%–82.0%), NPV of 69.72% (95% CI: 64.9%–74.5%), and diagnostic accuracy of 71.05% (95% CI: 67.1%–75.0%) for PCNSL diagnosis. Notably, the target sign was exclusive to PCNSL (14/14, 100%), resulting in 100% specificity and PPV. In terms of inter-rater agreement, excellent consistency was achieved for the purfling sign and tumor necrosis and cystic changes (κ= 0.982 and 0.869, respectively), with good agreement for parenchymal enhancement heterogeneity (κ= 0.709).

**Conclusion:**

Consistent with prior findings, the “purfling sign” serves as a valuable auxiliary diagnostic indicator for differentiating PCNSL from high-grade glioma, thereby facilitating improved diagnostic confidence and providing supportive evidence for clinical management.

## Introduction

Primary central nervous system lymphoma (PCNSL) and high-grade glioma are the two most common types of primary central nervous system (CNS) malignancies, yet their therapeutic strategies and clinical prognoses differ significantly [1–5]. PCNSL accounts for approximately 2–6% of all CNS malignancies [1, 4], with its incidence showing a gradual upward trend in recent years [1, 2]. Notably, PCNSL is highly sensitive to radiotherapy and chemotherapy, and combined chemoradiotherapy represents a standard first-line treatment strategy in clinical practice [1, 2, 6–8]. In contrast, glioma is the most prevalent primary malignant brain tumor, for which surgical resection forms the cornerstone of management, followed by standardized adjuvant therapy [1, 3, 9]. These divergent therapeutic paradigms highlight the critical importance of accurate preoperative differentiation between PCNSL and glioma—a misdiagnosis could lead to inappropriate treatment strategies and adverse patient outcomes [3, 9–11].

Conventional magnetic resonance imaging (MRI), including non-contrast and contrast-enhanced MRI (CE-MRI), serves as the primary imaging modality for the diagnosis and differential diagnosis of CNS tumors [1, 2, 9, 12, 13]. CE-MRI is commonly used in clinical practice due to its time efficiency, wide accessibility in primary care settings, and objective assessment of imaging signs. Over the past decade, the academic community has identified several characteristic imaging features of PCNSL, such as homogeneous enhancement, the “clenched fist sign”, “umbilicus sign”, “incision sign”, and the “sharp corner sign” [4, 14–16]. However, due to the inherent heterogeneity of PCNSL itself, PCNSL often exhibits atypical manifestations, leading to overlapping imaging findings with other CNS tumors—especially glioma [13]. This overlap poses considerable challenges to preoperative differential diagnosis, even for experienced neuroradiologists.

In recent years, the academic community has sought to identify specific imaging markers for improving the differential diagnostic accuracy of PCNSL. One novel CE-MRI feature, the “purfling sign”, has recently been proposed as a potential preoperative indicator for PCNSL [17]. However, this newly proposed sign has not yet gained widespread acceptance in the academic community and has not undergone rigorous systematic validation to confirm its reliability in distinguishing PCNSL from glioma. It remains unclear whether this sign is associated with the pathophysiological features of PCNSL or can serve as a stable and reliable imaging marker for differentiating the two malignancies.

Given the unmet clinical need for accurate preoperative differentiation between PCNSL and glioma, coupled with the lack of systematic validation for the purfling sign, we conducted a multicenter retrospective study across three centers. We aimed to evaluate the sign’s diagnostic value in differentiating PCNSL from glioma and assess its diagnostic performance and reproducibility as a potential imaging marker. This study seeks to provide evidence-based imaging support to improve preoperative diagnostic accuracy and support clinical decision-making.

## Methods

### Study population

We retrospectively screened a total of 618 patients with pathologically confirmed PCNSL or glioma from three medical centers between January 2010 and October 2025. After excluding 124 ineligible cases (non-enhancing parenchymal lesions: *n* = 62; prior radiochemotherapy: *n* = 23; poor image quality: *n* = 28; immunodeficiency-related lymphoma: *n* = 3; intravascular lymphoma: *n* = 8), 494 patients were finally enrolled and combined into a single large cohort for analysis. According to the pathological results, 209 cases were diagnosed with PCNSL and 285 with glioma (73 Grade 3, 212 Grade 4). All patients underwent CE-MRI, none of whom had received prior treatment before MRI scanning.

Patients were enrolled in this study based on the following inclusion criteria: (1) Aged ≥ 18 years, with lesions confined to the CNS and a confirmed pathological diagnosis; (2) Availability of pre-treatment CE-MRI datasets including conventional sequences-axial T1-weighted imaging (T1WI), T2-weighted imaging (T2WI), and contrast-enhanced T1-weighted imaging (CE-T1WI); (3) MR images free of motion artifacts or susceptibility artifacts that could affect diagnostic accuracy; (4) All PCNSL patients had normal immune function.

Exclusion criteria were as follows: (1) Diagnosis of intravascular lymphoma; (2) Glioma without obvious parenchymal contrast enhancement; (3) Prior treatment before MRI examination, including biopsy, surgical resection, radiotherapy, systemic chemotherapy, and corticosteroid therapy.

### MRI acquisition

All patients underwent MRI scanning using a 3.0T MRI scanner from different mainstream vendors equipped with a dedicated 16-channel head coil at three medical centers (center 1 used a Philips Ingenia CX 3.0T scanner; centers 2 and 3 used a Siemens Skyra 3.0T scanner). Patients were placed in a supine position with their heads secured and oriented perpendicular to the coil. Routine clinical sequences were acquired, including T1WI, T2WI, fluid-attenuated inversion recovery, and diffusion-weighted imaging. After intravenous administration of 0.1 mmol/kg gadolinium-diethylenetriamine pentaacetate at an injection rate of 2.5 mL/s, a CE-T1WI sequence was obtained in sagittal, coronal, and axial planes with the following parameters: FOV 220 × 220 mm, matrix 256 × 256, slice thickness 4 mm, flip angle 70°, TR 250 ms, TE 2.49 ms.

All imaging data were electronically stored in the standard picture archiving and communication system for subsequent review and analysis. Importantly, the core parameters of CE-T1WI were strictly consistent across all three centers, ensuring comparable image quality for evaluating the purfling sign. Although the MRI scanner manufacturers differed slightly among centers, this did not influence the identification and assessment of the target imaging features.

### Image evaluation

Two neuroradiologists with 6 and 10 years of experience in neuro-oncology imaging, who were blinded to patients’ histopathological diagnoses and clinical data, independently reviewed and assessed CE-T1WI features. Following established criteria from prior literature [17], the CE-T1WI features assessed included lesion number (single vs. multiple), purfling sign, necrosis and cystic changes, tumor parenchymal enhancement heterogeneity, and other related features. (1) The “purfling sign” was defined as well-demarcated, ring-enhanced lesions visualized at either the tumor margin or within the tumor parenchyma. This imaging feature is categorized into four distinct subtypes: (a) absent; (b) inner-ring purfling sign; (c) outer-ring purfling sign; and (d) combined inner-ring and outer-ring purfling sign, which is also designated as the target sign; (2)Tumor necrosis and cystic changes were classified into three grades: absent; mild, defined as necrosis and cystic comprising less than 50% of the entire tumor area; and extensive, where such pathological changes constituted 50% or more of the total tumor area; (3) Tumor parenchymal enhancement uniformity was classified into two categories: (a) homogeneous enhancement (uniform signal intensity throughout the tumor parenchyma); (b) heterogeneous enhancement (variable enhancement intensity across the tumor, including non-enhancing, mild, and marked enhancement relative to adjacent tissue). When discrepancies arose during feature assessment, such discrepancies were resolved via a consensus review conducted by a senior neuroradiologist with 20 years of experience in brain tumor diagnosis.

### Statistical analysis

All statistical analyses were performed using SPSS 25.0 software. Age, which exhibited a non-normal distribution, was compared between the PCNSL and glioma groups using the Mann-Whitney U test, and data were presented as median (P₂₅, P₇₅). Categorical data were expressed as frequencies (percentages) [n (%)]. The Chi-square (χ²) test was used to compare differences in the distribution of categorical variables between the two groups. Inter-rater agreement for lesion CE-MRI features between the two neuroradiologists was assessed using Cohen’s kappa (κ) coefficient. Agreement statistics were calculated based on the initial independent readings prior to consensus adjudication.

Consistency was graded as follows: κ ≤ 0.20, poor agreement; 0.21–0.40, fair agreement; 0.41–0.60, moderate agreement; 0.61–0.80, good agreement; and κ > 0.80, excellent agreement. A P-value < 0.05 was considered statistically significant for all analyses. To further evaluate the diagnostic performance of the “purfling sign” for PCNSL, its sensitivity, specificity, positive predictive value (PPV), and negative predictive value (NPV) were calculated based on the final consensus interpretations of the two neuroradiologists, with 95% confidence intervals (CIs) reported for all metrics.

## Results

### Cohort characteristics and patient enrollment

**Fig. 1:**
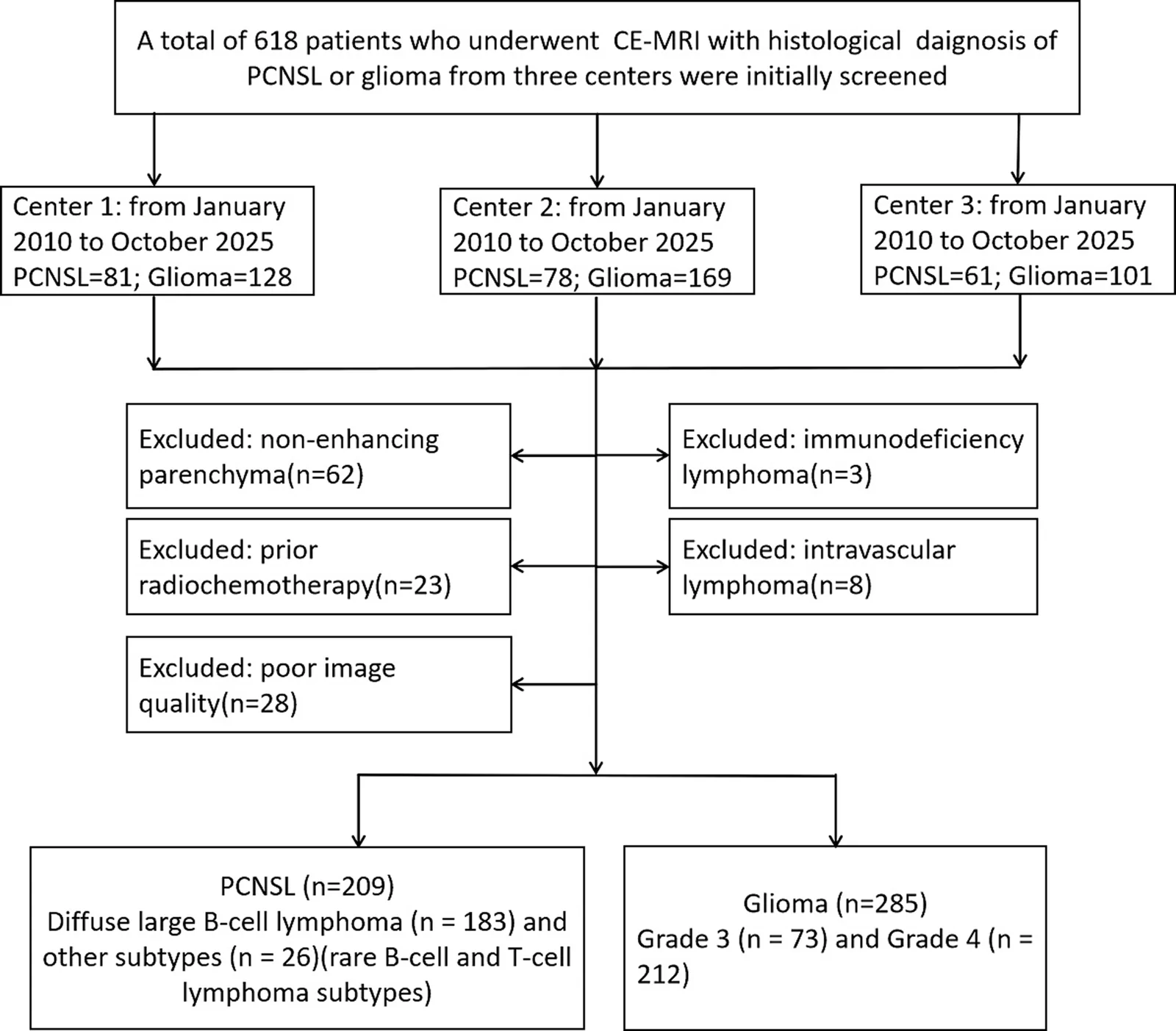
Flowchart of patient selection

Per the predefined inclusion and exclusion criteria, 209 patients with PCNSL and 285 with glioma from three centers were initially enrolled in this study. A total of 124 cases were excluded based on the exclusion criteria, including 3 PCNSL patients excluded due to known immunodeficiency, 8 cases of intravascular lymphoma, 62 glioma patients with non-enhancing parenchyma, 23 cases with a history of radiochemotherapy prior to admission, and 28 cases with imaging artifacts that precluded diagnostic assessment. A flowchart of patient selection is presented in Fig. 1.

Among the PCNSL cohort, 183 cases were diagnosed with diffuse large B-cell lymphoma, with the remaining 26 cases classified as other subtypes. For the glioma cohort, histopathological grading according to the World Health Organization (WHO) criteria revealed 73 cases of Grade 3 glioma and 212 cases of Grade 4 glioma.

### Reproducibility of neuroradiologist’s assessment

For the assessment of CE-T1WI features in 209 PCNSL and 285 glioma patients, all inter-observer agreement analyses were statistically significant (*P* < 0.001). Specifically, the kappa (κ) coefficients for the purfling sign and tumor necrosis and cystic changes were 0.982 and 0.869, respectively, both indicating excellent inter-observer agreement. For tumor parenchymal enhancement homogeneity, the kappa (κ) coefficient was 0.709, reflecting good inter-observer agreement (Table 1).

**Table 1:** Inter-observer agreement for CE-MRI features evaluation in PCNSL and glioma patients

Imaging Features	Kappa	*P*	Agreement level
Purfling sign	0.982	0.000	excellent
Lesion necrosis and cystic changes	0.869	0.000	excellent
Parenchymal enhancement heterogeneity	0.709	0.000	good

### Comparison of CE-MRI features between PCNSL and Glioma

**Fig. 2:**
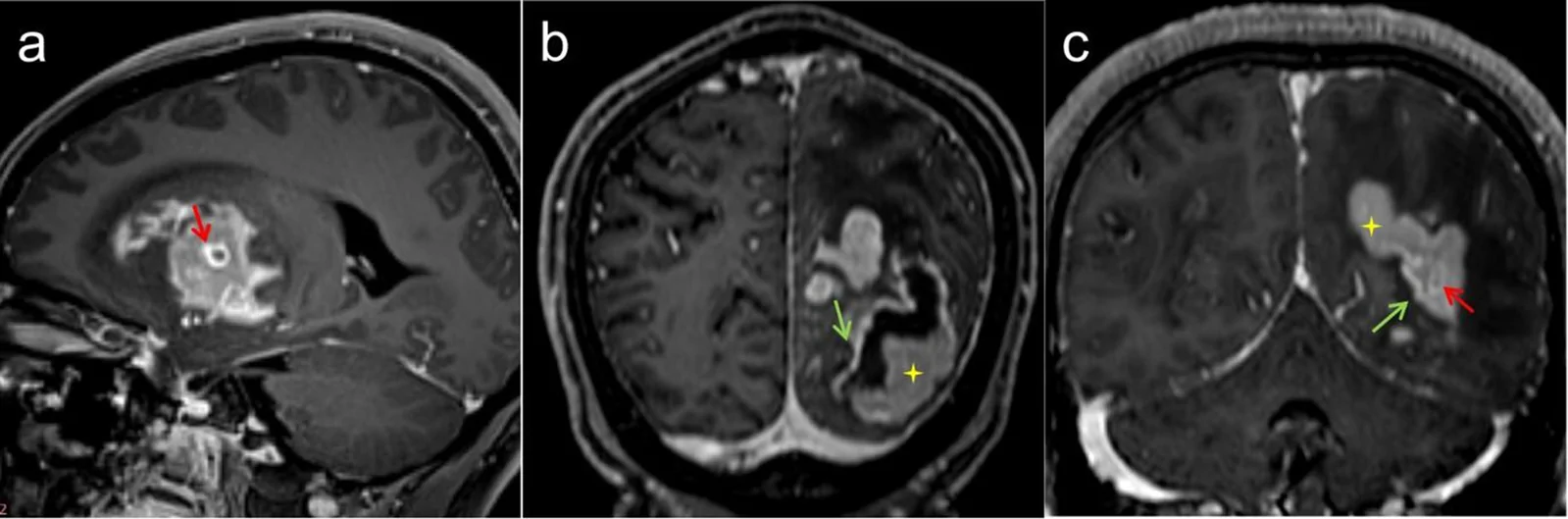
Purfling sign subtypes in PCNSL. **a-c** Multiple lesions of PCNSL, diffuse large B-cell lymphoma. **a** inner-ring purfling sign (red arrow). **b** outer-ring purfling sign (green arrow). **c** target sign (green arrow and red arrow). **b-c** homogeneous parenchymal enhancement (yellow star)

**Fig. 3:**
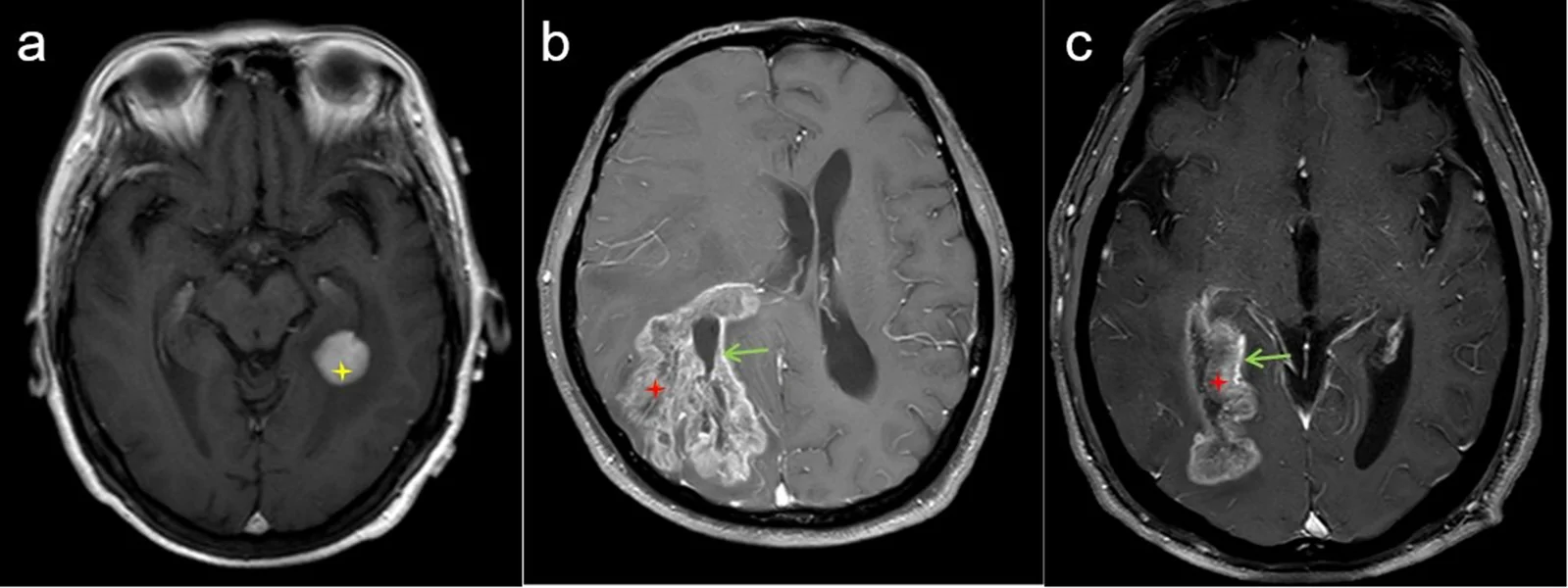
Imaging features in PCNSL and glioma. **a** Single lesion of PCNSL, diffuse large B-cell lymphoma: homogeneous parenchymal enhancement (yellow star). **b-c** Single lesion of glioma, outer-ring purfling sign (green arrow); heterogeneous parenchymal enhancement (red star)

Demographic and imaging features of the 209 PCNSL patients and 285 glioma patients are summarized in Table 2. No significant differences were observed in age (*P* = 0.360) or sex distribution (*P* = 0.827) between the two cohorts. Lesion number differed significantly between groups (*P* = 0.001): single lesions were more common in glioma patients (70.9% vs. 56.0% in PCNSL), while multiple lesions were more frequent in PCNSL (44.0% vs. 29.1% in glioma). CE-T1WI findings revealed a significant difference in the purfling sign between PCNSL and glioma (*P* < 0.001). Specifically, the inner-ring purfling sign (18.7% in PCNSL vs. 3.9% in glioma) (Fig. 2A), the outer-ring purfling sign (22.5% in PCNSL vs. 8.1% in glioma) (Fig. 2B), and the target sign (6.7% in PCNSL vs. 0% in glioma) (Fig. 2C) were all more prevalent in PCNSL patients. Lesion necrosis and cystic changes differed markedly (*P* < 0.001): no changes (61.2% in PCNSL vs. 9.1% in glioma) and mild changes (34.9% in PCNSL vs. 19.3% in glioma) were more common in PCNSL, while obvious changes dominated in glioma (71.6% in glioma vs. 3.8% in PCNSL). Parenchymal enhancement heterogeneity also varied significantly (*P* < 0.001): homogeneous enhancement was more common in PCNSL (77.0% vs. 47.4% in glioma) (Fig. 3A), while heterogeneous enhancement was more frequent in glioma (52.6% vs. 23.0% in PCNSL) (Fig. 3B and C).

**Table 2:** Demographic and CE-MRI features of patients with PCNSL and glioma

Parameters	Lymphoma (*n* = 209)	Glioma (*n* = 285)	t/z/χ2	*P*
Age (y), Median (P_25_, P_75_)	58(54.0, 69.5)	58(54.0, 68.5)	−0.915	0.360
Sex, n (%)			0.048	0.827
Male	116(55.5)	161(56.5)		
Female	93(44.5)	124(43.5)		
Number of lesions, n (%)			11.696	0.001
Single	117(56.0)	202(70.9)		
Multiple	92(44.0)	83(29.1)		
Purfling sign (%)			-	<0.001
Inner ring	39(18.7)	11(3.9)		
Outer ring	47(22.5)	23(8.1)		
Target sign	14(6.7)	0(0.0)		
Lesion necrosis and cystic changes (%)			245.414	<0.001
None	128(61.2)	26(9.1)		
Mild	73(34.9)	55(19.3)		
Obvious	8(3.8)	204(71.6)		
Parenchymal enhancement heterogeneity (%)			44.183	<0.001
Homogeneous	161(77.0)	135(47.4)		
Heterogeneous	48(23.0)	150(52.6)		

### Diagnostic efficacy of CE-MRI features

The diagnostic performance of CE-T1WI imaging features for differentiating PCNSL from glioma was quantified by sensitivity, specificity, PPV, NPV, and accuracy. For the purfling sign, its diagnostic metrics for identifying PCNSL were as follows: sensitivity of 47.85% (95% CI: 41.1%–54.6%), specificity of 88.07% (95% CI: 84.3%–91.8%), PPV of 74.63% (95% CI: 67.3%–82.0%), NPV of 69.72% (95% CI: 64.9%–74.5%), and diagnostic accuracy of 71.05% (95% CI: 67.1%–75.0%). As a subtype of the purfling sign, the target sign was exclusively detected in 14 PCNSL cases (4.91%) and was absent in all glioma patients; its corresponding values for differential diagnosis were 6.70% sensitivity, 100.00% specificity, and 100.00% PPV, thus identifying it as a highly specific imaging marker for PCNSL.

## Discussion

The core finding of this study confirms that the “purfling sign”—distinct from conventional PCNSL-associated imaging manifestations such as the “clenched fist sign”, “umbilication sign”, and “spiculated sign”—acts as a useful imaging marker for differentiating PCNSL from glioma, which is consistent with the findings of existing relevant research [17]. Specifically, a prior study verified that among 114 tumors positive for the “purfling sign”, 97 cases (85.09%) were PCNSL and 17 cases (14.91%) were glioma; subsequent statistical analysis demonstrated a significant association between this sign and PCNSL (*p* < 0.001), with diagnostic parameters including 90.40% specificity, 54.80% sensitivity, 85.09% PPV, and 66.67% NPV [17].

Consistent with these previous findings [17], our study demonstrated that the purfling sign achieved a sensitivity of 47.85%, specificity of 88.07%, PPV of 74.63%, NPV of 69.72%, and diagnostic accuracy of 71.05% for differentiating PCNSL from glioma. Notably, its incidence in the PCNSL group (47.8%) was significantly higher than that in the glioma group (8.4%), which is consistent with the study [17], further enhancing its practical diagnostic utility. Regarding the “target sign”, both previous literature [17] and our study confirmed its exceptional diagnostic performance: all target sign positive lesions were PCNSL (literature: 25/25, 100%; our study: 14/14, 100%), with the sign completely absent in all glioma cases, yielding 100% specificity and PPV. Although both the purfling sign and target sign have relatively low sensitivity (literature: 5.08% for target sign; our study: 6.70% for target sign in PCNSL cases), their presence may provide supportive supplemental evidence for the differentiation of PCNSL from glioma, especially in diagnostically challenging cases with overlapping imaging features.

While lesion necrosis and cystic changes exhibited marginally higher diagnostic accuracy for this differentiation, this feature lacks clinical disease specificity in clinical practice. As common imaging findings of intracranial malignant tumors with high proliferative activity and insufficient blood supply (e.g., glioblastoma, brain metastases) [18–20], necrosis and cystic changes are not specific to PCNSL. Moreover, the unique 100% specificity of the target sign further highlights the irreplaceable role of the purfling sign system in the diagnosis of PCNSL. In clinical settings where necrosis and cystic changes are absent or equivocal, the presence of the purfling sign can offer valuable supplementary diagnostic evidence for confirming PCNSL, thereby confirming that the purfling sign is a highly specific auxiliary imaging feature that complements the general diagnostic utility of necrosis and cystic changes in the differential diagnosis of intracranial tumors.

Consistent with prior studies [1, 2, 17, 21–23], three conventional imaging features— tumor lesion number, tumor necrosis and cystic changes, and tumor parenchymal enhancement heterogeneity aided in differentiating PCNSL from glioma: PCNSL typically presents as multiple lesions [1, 2, 4, 17, 22], with no or mild tumor necrosis and cystic changes and homogeneous enhancement, while high-grade glioma tends to be single lesion with severe necrosis and cystic changes and heterogeneous enhancement. Combining these features with the purfling sign synergistically improves diagnostic efficiency, which is critical for optimizing treatment decisions. Given their distinct therapeutic paradigms (PCNSL: first-line chemoradiotherapy; gliomas: first-line surgical resection), accurate preoperative differential diagnosis facilitates personalized treatment planning, avoiding unnecessary surgical trauma in PCNSL patients while ensuring timely intervention for glioma patients.

The distinct CE-MRI features between PCNSL and glioma stem fundamentally from their inherent pathological differences. For PCNSL, homogeneous enhancement originates from its unique hypovascularity and perivascular‑cuff‑like infiltration pattern of diffuse large B-cell lymphoma cells. Tumor cells spread along intracranial vascular walls, damage endothelial cells, and disrupt the blood-brain barrier (BBB) uniformly, leading to consistent contrast leakage and homogeneous parenchymal enhancement [8, 14, 24]. Regarding the purfling‑sign subtypes, the inner‑ring sign arises from central hypoxia‑induced lymphoma‑cell apoptosis, which forms a non‑enhancing necrotic core surrounded by inflammatory‑mediated perinecrotic enhancement. The outer‑ring sign results from continuous peripheral perivascular infiltration and regular BBB disruption, presenting as a smooth ring‑shaped enhancement [8, 24]. The target sign combines both central apoptosis and peripheral infiltration mechanisms, explaining its high specificity for PCNSL. These potential mechanisms are speculative and require further histopathologic validation.

In contrast, high‑grade gliomas show marked pathological heterogeneity. Hypoxia‑driven neovascularization via the hypoxia-inducible factor-vascular endothelial growth factor pathway generates disorganized, immature blood vessels with defective BBB function. Regional variations in BBB integrity and vascular distribution cause heterogeneous contrast enhancement throughout the tumor parenchyma [25–27].

Moreover, glioma‑driven rapid proliferation with insufficient blood supply leads to extensive micro‑necrosis, which gradually coalesces into large cystic necrotic areas with irregular enhancement rims. This pathological heterogeneity prevents the formation of continuous regular ring‑shaped enhancement, so the purfling sign is rarely observed in glioma tissues [28].

Additionally, PCNSL rarely presents with large‑scale cystic necrosis due to its balanced proliferation‑apoptosis regulation and hypovascular nature. Insufficient nutrition mainly induces cell migration rather than massive necrosis, further distinguishing it from glioma [27].

Regarding lesion number, PCNSL tends to present with multiple lesions more frequently than high-grade glioma, a feature that reflects the divergent invasive patterns of the two tumor types. PCNSL cells exhibit high motility and migrate along the intracranial vascular network to form multiple lesions. In contrast, high-grade gliomas exhibit predominantly local growth, with multiple lesions rarely occurring. When multifocality develops in glioma, it is typically secondary to cerebrospinal fluid dissemination or contiguous invasion, reflecting their limited long-distance migratory capacity [29].

Furthermore, excellent inter-observer agreement in identifying the purfling sign and other evaluated features (lesion number, tumor cystic necrosis, tumor parenchymal enhancement heterogeneity) validates their robust reproducibility on CE-T1WI. This reproducibility derives from the distinct, characteristic morphological presentations of these signs—particularly the PCNSL specific target sign—and underscores their translational potential for clinical practice: it ensures consistent evaluation across radiologists and institutions, bolsters the robustness of diagnostic inferences, and furnishes a solid foundation for informing clinical treatment stratification.

This study has several limitations that should be acknowledged. First, despite being a multicenter retrospective study involving three institutions, the sample size was relatively small, which may have introduced selection bias and compromised the generalizability of the results. Future large-cohort multicenter prospective studies are needed to validate the conclusions. Second, other intracranial tumors (e.g., meningiomas, brain metastases) that may occasionally present with the purfling sign were not included, restricting the comprehensive evaluation of the sign’s differential diagnostic value across intracranial tumors; its utility in distinguishing PCNSL from these tumors requires further exploration. Third, only CE-MRI features were evaluated, with no integration of multi-modal MRI sequences (e.g., diffusion-weighted imaging, perfusion-weighted imaging) or molecular markers for comprehensive analysis. Combining these modalities and markers is expected to further improve the differential diagnostic accuracy between PCNSL and glioma. In addition, the purfling sign may be less conspicuous in small or deep lesions, which warrants validation in larger cohorts. Furthermore, potential inter-observer variability in visual assessment might be reduced in future studies with artificial intelligence-aided recognition.

## Conclusion

The purfling sign on CE-MRI is a valuable auxiliary imaging indicator that demonstrates high specificity for PCNSL. It is straightforward to identify on routine CE-MRI and shows excellent inter-observer reproducibility, making it applicable even in primary healthcare institutions. This sign improves the accuracy of preoperative differential diagnosis between PCNSL and high-grade glioma, supports optimal therapeutic strategy selection, and avoids unnecessary surgical trauma. Given its prominent diagnostic value and favorable applicability, the “purfling sign” has promising clinical application value as a valuable adjunctive diagnostic indicator for PCNSL. Finally, pathological diagnosis remains the gold standard, and the purfling sign serves only as an auxiliary imaging indicator.

## Data Availability

The raw clinical imaging and patient data generated and analyzed during this study are not publicly available due to patient privacy protection. Data can be obtained from the corresponding author upon reasonable request.

## Abbreviations

BBB: Blood-Brain Barrier
CE-MRI: Contrast-Enhanced Magnetic Resonance Imaging
CE-T1WI: Contrast-Enhanced T1-Weighted Imaging
CNS: Central Nervous System
DLBCL: Diffuse Large B-Cell Lymphoma
NPV: Negative Predictive Value
PCNSL: Primary Central Nervous System Lymphoma
PPV: Positive Predictive Value
WHO: World Health Organization
